# The prevalence of anatomical variants of the coeliac trunk and renal arteries on contrast-enhanced abdominal computed tomography scans at Dr George Mukhari Academic Hospital

**DOI:** 10.4102/sajr.v25i1.1990

**Published:** 2021-01-25

**Authors:** Raeesa Omar, Margaret Kisansa, Alireza D. Dehnavi

**Affiliations:** 1 Department of Diagnostic Radiology & Imaging, Faculty of Health Sciences, Dr George Mukhari Academic Hospital, Sefako Makgatho Health Sciences University, Pretoria, South Africa

**Keywords:** anatomical, variants, coeliac, trunk, renal, arteries, CT, scan

## Abstract

**Background:**

Anatomical variations of the coeliac trunk and renal arteries should be radiologically reported as they affect the surgical approach and subsequent outcome in patients.

**Objectives:**

The aim of this study was to determine the prevalence of anatomical variations of the coeliac trunk and renal arteries and whether there is a relationship between the occurrence of these variations at Dr George Mukhari Academic Hospital.

**Method:**

Arterial phase abdominal computed tomography (CT) or CT abdominal angiograms performed during January and December 2017 were analysed. The variations of the coeliac trunk and renal arteries were classified according to accepted classification systems and expressed as a percentage of the study population.

**Results:**

A normal classical coeliac trunk was present in 82.2% and a non-classical pattern was present in 9.7%. The most common variation of the coeliac trunk other than the non-classical pattern was a hepatosplenic trunk, which was present in 3% of the study population. A normal right and left renal artery was present in 88.2% and 83.7%, respectively. The most common variations of the renal arteries were bilateral hilar arteries seen in 3.4% on the right and 9.1% on the left. Renal artery variations were more prevalent on the left than on the right. Concurrent variations of both the right and the left renal arteries were present in 2.4% and variations of both the coeliac trunk and renal arteries were present in 5% of the study population.

**Conclusion:**

The most common variation of the coeliac trunk in this study is comparable to other studies in non-African populations. Concurrent vascular variations between the renal arteries and between the coeliac trunk and renal arteries may co-exist.

## Introduction

Vascular variations in the coeliac trunk and renal arteries are commonly encountered. These variations directly affect the surgical approach, should patients undergo surgical interventions such as organ transplantation or organ/tumour resection. It is therefore important to recognise these variations and give an accurate concise description thereof in the report when assessing a contrasted abdominal computed tomography (CT) scan or a CT angiogram of the abdominal aorta.

Several studies, which signified the importance of identifying these variations, have been analysed, and they emphasise the importance of correctly reporting upon these variations as a radiologist. The identification and correct classification of these variations aid in the subsequent management and may result in more favourable surgical outcomes.

The coeliac trunk is the first major branch of the abdominal aorta and arises anteriorly from the abdominal aorta at the level of T12 behind the median arcuate ligament. Normal coeliac trunk anatomy includes the division of the coeliac trunk into three branches – the common hepatic artery (CHA), the left gastric artery and the splenic artery. The left gastric artery is usually the first branch, after which the coeliac artery bifurcates into the splenic artery and the CHA. This is known as the classic trifurcation.^1,2^

The aorta is connected to the ventral longitudinal anastomosis by the 10th, 11th, 12th and 13th vitelline arteries in primitive vasculature. Usually, the coeliac trunk and the superior mesenteric artery are formed by the 10th and 13th vitelline arteries with the remaining segments regressing before birth. Variants arise when this process does not occur in the prescribed order. For example, if the 10th and 12th vitelline arteries regress but there is abnormal persistence of the ventral anastomoses, a coeliaco-mesenteric trunk occurs.^3^

**FIGURE 1:**
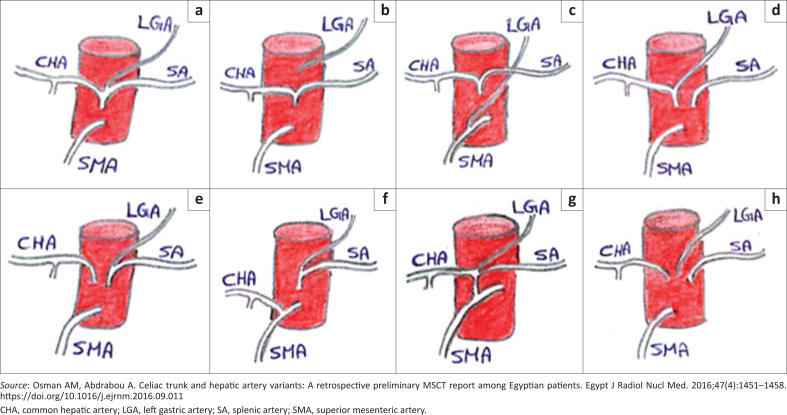
Schematic representation of the different types of coeliac trunk variants according to Ulflacker’s classification: (a) type I; (b) type II; (c) type II; (d) type III; (e) type V; (f) type V; (g) type VI; (h) type VIII.

**FIGURE 2:**
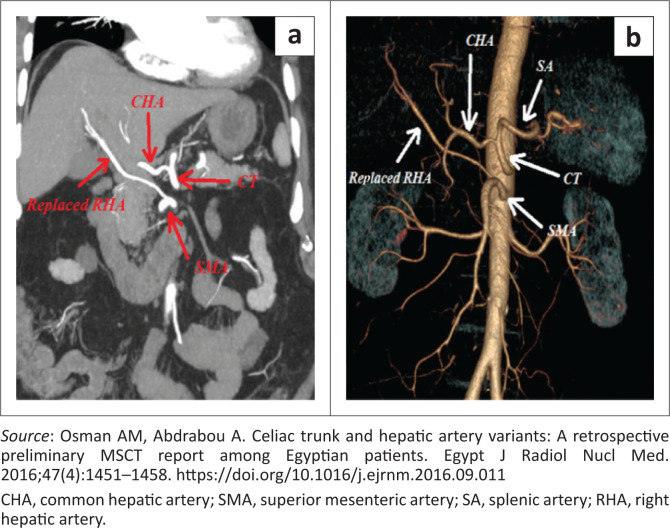
Type 1 classic trifurcation. Coronal multi-planar reconstruction image (a) and three-dimensional reconstruction images (b) show a type I coeliac classic trifurcation. Additionally, there is a replaced right hepatic artery.

**FIGURE 3:**
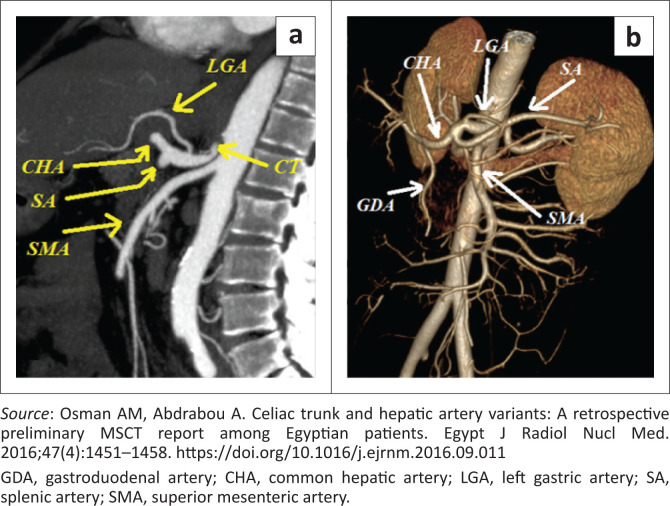
Sagittal multiplanar reconstruction (a) and three-dimensional (b) images show Ulflacker’s Type 1 non-classic pattern.

In the literature, a variable number of variations of the coeliac trunk have been documented. For ease of reference, Ulflacker’s classification will be used to classify the type of variation encountered (Table 1, Figure 1).^4^ This classification was chosen as it is simple but provides adequate detail of each variant type. According to the classification in Table 1, eight types of coeliac trunk variations have been classified thus far. An example of a type 1 classic trifurcation is illustrated in Figure 2 and a non-classic trifurcation is illustrated in Figure 3 by Osman et al.^4^

**TABLE 1:** Ulflacker’s classification.

Classification	Description
**Type I = trifurcation**	
Classic pattern	The CHA, SA and LGA have a common point of origin from the coeliac trunk
Non-classic pattern	CHA and SA have a common point of origin with the LGA demonstrating variable points of origin
Type II = hepato-splenic trunk	CHA and SA have a common trunk with the LGA arising separately from aorta
Type III = hepato-gastric trunk	CHA and LGA have a common trunk with the SA arising separately from the aorta or SMA
Type IV = hepato-spleno-mesenteric trunk	CHA, SA and SMA have a common trunk with the LGA arising separately from the aorta
Type V = gastro-splenic trunk	LGA and SA have a common trunk with the CHA arising separately from the aorta or SMA
Type VI = coeliaco-mesenteric trunk	Coeliac and SMA have a common trunk
Type VII = coeliaco-colic trunk	The middle colic artery and the coeliac have the same trunk
Type VIII = no coeliac trunk	No coeliac trunk with the CHA, SA and LGA arising directly from the aorta

*Source*: Osman AM, Abdrabou A. Celiac trunk and hepatic artery variants: A retrospective preliminary MSCT report among Egyptian patients. Egypt J Radiol Nucl Med. 2016;47(4):1451–1458. https://doi.org/10.1016/j.ejrnm.2016.09.011

CHA, common hepatic artery; LGA, left gastric artery; SA, splenic artery; SMA, superior mesenteric artery.

The anatomy of the coeliac trunk was found to be highly variable when analysed in a number of different studies. The CT scans and digital subtraction angiograms (DSAs) of 5002 patients were retrospectively analysed by Song et al.^5^, in Seoul, South Korea, who found that 89.1% of these patients had normal coeliac trunk anatomy represented by a hepatogastrosplenic trunk (includes classic and non-classic trifurcation). In 9.64% of the patients, 12 specific types of variations of the coeliac trunk were identified. The study further redefined the CHA as an arterial trunk from which at least one segmental hepatic artery and the gastroduodenal artery originates from, regardless of the origin or path of this trunk.

In another study by Prakash et al.^6^ in India, variations in the coeliac trunk were studied in 50 cadavers. Of these, 86% demonstrated normal coeliac trunk anatomy with the classic trifurcation. The most common pattern observed in 76% of subjects was that of the left gastric artery originating from the coeliac trunk proximal to the bifurcation into the common hepatic and splenic arteries.^6^ A further study performed on cadavers by Chitra et al, found that the branching pattern of the coeliac trunk varied from the classic trifurcation to an abnormal trifurcation and even to four, five and six branching patterns of the coeliac trunk.^7^

When classifying the different types of hepatic artery variations according to the Michels and Hiatt classification (which classifies hepatic artery variations into 10 and 6 types, respectively), it was found that normal anatomy was found in 79.1% of patients, whilst variant or anomalous anatomy was found in 20.9% of patients. The authors concluded that identification of the variant types were vital in the pre-operative and intra-operative planning and technique and was thus of importance to both the radiologist and the surgeon.^8^

The identification of variants of the coeliac axis was shown to be important prior to surgical procedures such as liver transplantation or angiographic procedures, and knowledge thereof is thus necessary in preventing intra-operative complications. Furthermore, knowledge of variations of the coeliac trunk is essential to ensure correct vascular anastomosis during liver transplant surgery, as well as in surgeries involving the pancreas, stomach and duodenum.^5,6^

Coeliac trunk variations are also of importance during endovascular intervention such as coeliac artery embolisation, which may be performed in patients with coeliac artery aneurysms or in repair of thoracoabdominal aortic aneurysms in the region of the coeliac artery. Studies have shown that certain variations such as a type IV or type VI trunk, wherein the coeliac artery shares an origin with the superior mesenteric artery, make coeliac artery embolisation impossible. Thus, it is essential for the vascular surgeon to be made aware if such variations exist.^9^

Interestingly, a correlation between the presence of accessory renal arteries and a higher incidence of variations in the coeliac and/or hepatic arteries in patients was found. Urgurel et al. ^10^, assessed variations in the hepatic arteries, coeliac trunk and renal arteries in 100 patients on multidetector CT angiography. In 89% of these patients, normal coeliac trifurcation was found in 48% of the patients also demonstrating hepatic artery variation. However, coeliac trunk and/or hepatic artery variation was found in 39.7% of the 58 patients with normal renal arteries and in 64.3% of the 42 patients with accessory renal arteries. There was thus a significant correlation between variations in the coeliac trunk and/or hepatic arteries and variations in the renal arteries. It can therefore be assumed that when an anatomic variation in the vascular supply of a single organ system is encountered, one should be mindful that anatomical variation of the vascular supply to other organ systems may co-exist.

The renal arteries usually arise as branches of the abdominal aorta below the level of the superior mesenteric artery. Normal renal arterial anatomy comprises a single renal artery supplying each kidney. Beregi et al^11^. found that in the vast majority of the 100 patients in whom spiral CT angiograms were assessed, the left (87%) and the right (88%) renal arteries originated between the lower border of the L1 and lower border of the L2 vertebral bodies. The right renal was found to most frequently originate at the lower border of L1 and the left renal artery at the L1/L2 intervertebral disc space.

The renal arteries are located anterior to the renal pelvis and enter each kidney at the medial aspect of the hilum. The renal veins are located posterior to the renal arteries. The right renal artery has a more prominent downward course and propagates behind the inferior vena cava to enter at the hilum of the more inferiorly located right kidney, whilst the left renal artery has a more prominent horizontal course to enter at the hilum of the superiorly located left kidney.^12^

**FIGURE 4:**
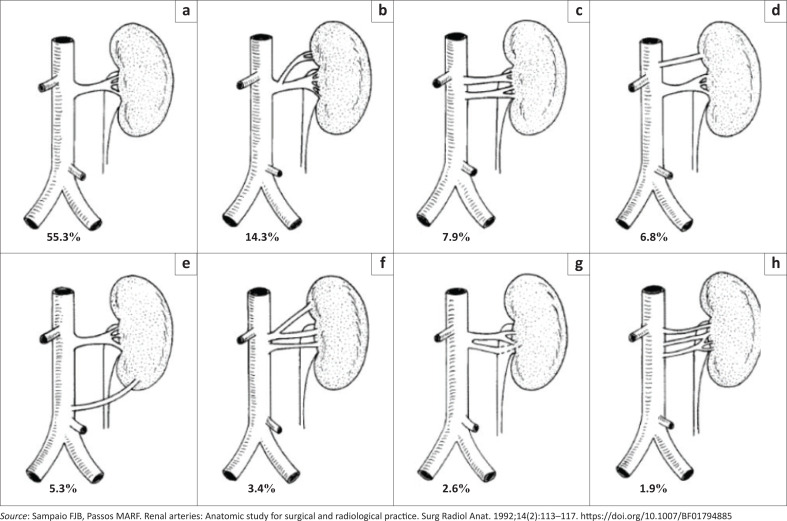
Variations in renal arteries: (a) type 1a – 1 hilar artery, (b) type 1b – 1 hilar with 1 superior pole extra-hilar branch, (c) type 1c – 2 hilar arteries, (d) type 1d – 1 hilar with 1 superior polar artery, (e) type 2a – 1 hilar with 1 inferior polar artery, (f) type 2b – 2 hilar arteries with 1 superior pole extrahilar branch, (g) type 2c – 1 hilar with a precocious bifurcation, and (h) type 2d – 3 hilar arteries.

**FIGURE 5:**
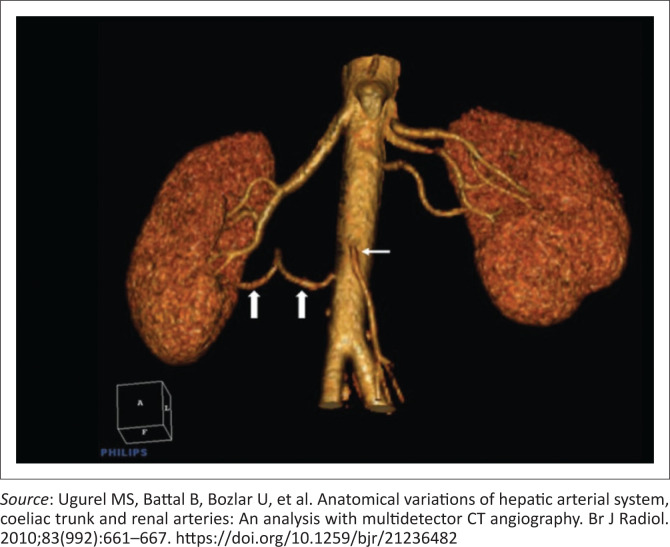
Accessory renal arteries – three-dimensional volume rendered image shows two renal arteries on the right – there is an accessory inferior polar artery (thick arrows) arising from the aorta below the inferior mesenteric artery (thin arrow). There are three renal arteries on the left.

In a study by Sampaio et al.^13^, in Brazil, the renal arteries of 266 kidneys of 133 subjects were dissected and analysed in order to illustrate the different variations (Figure 4). The variations depicted in Figure 4 will be used in the classification and description of the type of renal artery variation. A three-dimensional CT reconstructed image of accessory renal arteries as depicted by Urgurel et al.^10^ is demonstrated in Figure 5.

It is important to identify variations in the renal vasculature especially in surgical procedures such as renal transplantation, nephrectomy, surgical treatment of renal artery stenosis or abdominal aortic aneurysm repair.^14^ The most common cause of renal artery stenosis is atherosclerosis followed by fibromuscular dysplasia. The surgical treatment of renal artery stenosis includes endovascular stenting of the renal arteries.^12^ Multidetector CT provides crucial information of the vasculature of interest and can thus be used as an alternative to invasive procedures such as conventional angiography for pre-operative planning of surgical procedures.^15^ Multidetector CT is a thus a reliable, non-invasive method to assess the anatomy and possible variations of the renal vasculature.

The above studies highlight the importance of identifying the vascular variations of the coeliac-hepatic axis together with that of the renal arteries. It has been observed that registrars in the department of Diagnostic Radiology at Dr George Mukhari Academic Hospital often fail to mention the vascular variants encountered in the coeliac trunk and renal arteries when reporting on CT scans or CT angiograms. This omission negatively affects any surgical intervention that may follow when the indication of the CT scan is one which would aid in such intervention.

This study was conducted to assess the prevalence of the vascular variations of the coeliac trunk and renal arteries expressed as a percentage of the study population and to determine whether there was a significant relationship between the occurrence of these variations. This study emphasises the importance of radiologists reporting on these variations to better aid the surgeon in the subsequent surgical management at Dr George Mukhari Academic Hospital.

## Methods

This was a retrospective, descriptive and quantitative study. Data was collected for the period January 2017 to December 2017. A sample size of 312 cases was initially calculated based on estimation of the average number of abdominal CT scans/CT angiograms done per week at Dr George Mukhari Academic Hospital.

The inclusion criteria included male and female adult patients above the age of 18 years old who had undergone a contrast-enhanced CT abdomen – specifically an arterial phase CT or a CT angiogram of the abdominal aorta during the study time period. The exclusion criteria included patients below 18 years of age, as image quality is reduced in paediatric patients because of a lower radiation dose.

The coeliac trunk and renal arteries were analysed on multi-planar reconstruction (MPR) and maximum intensity projection (MIP) images (axial, coronal and sagittal views) with a slice thickness of 10 mm on MIP images. This was acquired on the Carestream picture archiving and communication system (PACS) in the Department of Radiology at Dr George Mukhari Academic Hospital.

The scans were analysed separately by two individual readers. The readers included a senior radiology registrar and a junior consultant radiologist. The readings were then reviewed and compared for congruency. Incongruent findings were identified and then subjected to analysis by a third reader who is a senior consultant radiologist. In order to eliminate bias, the third reader performed the readings blindly without knowledge of the other readers’ findings. In these cases, readings were considered valid and included in the results if congruency was established between two of the three readers.

Of the starting 312 cases, a total of 301 cases were adequate and selected for analysis. Furthermore, three cases were omitted because of the lack of consensus between the readers. Because of the discrepancies amongst the readers, the sample sizes were then adjusted to represent each vessel as follows: coeliac trunk - 298 cases; right renal artery - 297 cases; and left renal artery - 296 cases.

All statistical analyses were performed on SAS statistical analysis software (SAS Institute Inc, Carey, NC, USA), Release 9.4.

### Ethical consideration

Ethical clearance was received from Sefako Makgatho Health Sciences University Research Ethics Committee (SMUREC). Ethical clearance number: SMUREC/M/280/2018: PG.

## Results

**FIGURE 6:**
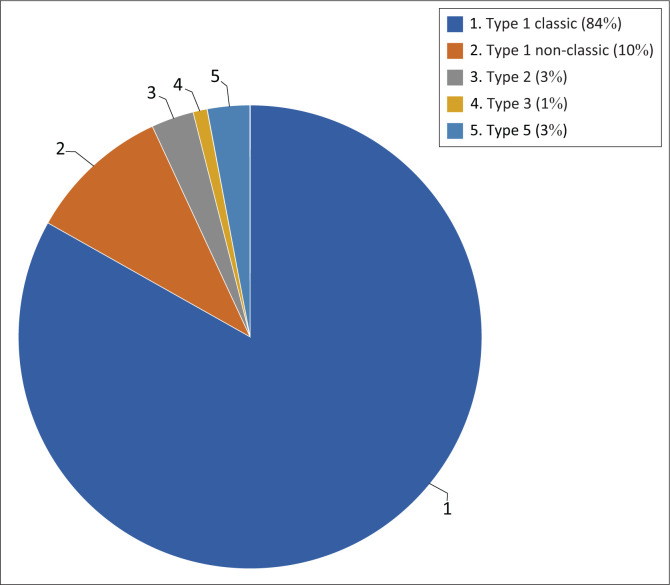
Coeliac trunk variations: The most common variant types are expressed as a percentage.

Of the study population, 82.2% demonstrated the classic type I coeliac trunk (normal anatomy) with 9.7% of the study population demonstrating type I non-classic coeliac trunk anatomy (*p* < 0.001). Furthermore, 3% of the study population demonstrated a type II (hepatosplenic trunk) anatomic configuration (*p* < 0.001), 1% of the study population demonstrated a type III (hepatogastric trunk) and 2.7% of the study population demonstrated a type V (gastrosplenic trunk) anatomy (*p* < 0.001) (Figure 6). The most common variant of the coeliac trunk other than the non-classical pattern was a type II (hepatosplenic trunk) closely followed by a type V (gastrosplenic trunk) anatomic configuration. The occurrence between a type II and type V was statistically insignificant (*p* = 1). The other coeliac trunk variations were either not present or, if present, observed in less than or equal to 1% of the study population.

**FIGURE 7:**
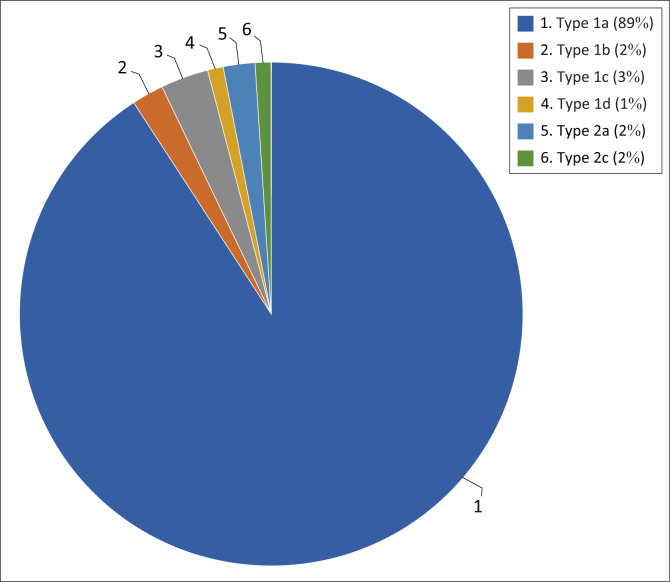
Right renal artery variations: The most common variant types are expressed as a percentage.

The anatomic configuration of the right renal artery was observed in the study population as follows: A type 1a (normal anatomy) right renal artery was seen in 88.2%; type 1b in 2.3%; type 1c in 3.4%; type 1d in 1.3%; type 2a in 2% and type 2c in 2.3% of the study population. The most common variant of the right renal artery was therefore a type 1c (2 hilar arteries) anatomy (*p* < 0.001). Other right renal artery variations were not observed in this study (Figure 7).

**FIGURE 8:**
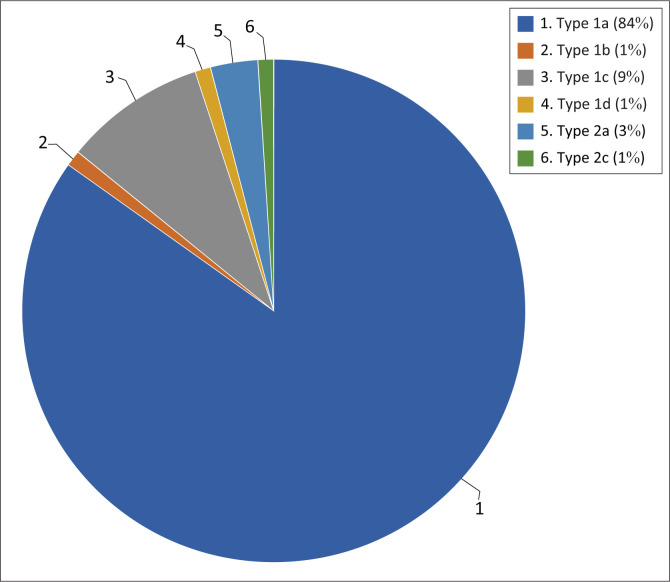
Left renal artery variations: The most common variant types are expressed as a percentage.

The anatomic configuration of the left renal artery was observed in the study population as follows: A type 1a (normal anatomy) left renal artery was seen in 83.7%; type 1b in 1%; type 1c in 9.1%; type 1d in 1.3%; type 2a in 2.7% and type 2c in 1.3% of the study population. The most common variant of the left renal artery was therefore a type 1c (2 hilar arteries) anatomy (*p* < 0.001). The other left renal artery variations were either not present or, if present, observed in less than 1% of the study population (Figure 8).

Concurrent variations of both the left and the right renal arteries were observed in 2.4% of the investigated cases. Further, variations of the coeliac trunk associated with either variations of the left renal artery, right renal artery or bilateral renal arteries were present in 5% of the investigated cases (95% confidence interval is 3.1% – 8.1%.). This shows that there might be a correlation between variations of the coeliac trunk and variations of the renal arteries, and further investigation may be warranted with future studies.

## Discussion

The importance of anatomical variations of the coeliac trunk and renal arteries and the importance of accurate radiological assessment and relevant reporting thereof lie in the surgical implications of such variations. These variations are often overlooked and are not routinely reported at Dr George Mukhari Academic Hospital.

In a study comparing findings from pre-operative CT angiography to intra-operative findings, it was observed that the correlation of anatomical similarities between the CT findings and the intra-operative findings was 98%.^16^ This reiterates that CT scan is an excellent modality for the assessment of the vascular anatomy. It is also postulated that anatomical variations may also be the source of certain pathological conditions such as vascular compression syndromes, which once again highlights the importance of identifying anatomical variations through the use of imaging modalities.^17^

In this study population, the most common variant of the coeliac trunk other than the non-classic pattern was a type II (hepatosplenic trunk) closely followed by a type V (gastrosplenic trunk) configuration. This is similar to the findings from a study conducted by Iezzi et al^18^. in Italy published in 2008, which also documented that a type II (hepatosplenic trunk) was the most common variation of the coeliac trunk.

Various other studies have indicated that a type V (gastrosplenic trunk) was the most common variant of the coeliac trunk as summarised later. In the study conducted by Arifuzzaman et al.^19^, a type V (gastrosplenic trunk) was the most common variant of the coeliac trunk followed by a type III (hepatogastric trunk) in the study population at the Dow Institute of Radiology in Pakistan whilst Torres et al.^20^ established that a type V (gastrosplenic trunk) was the most common variant of the coeliac trunk in the study population at a Lublin hospital in Poland. Lastly, Urgurel et al^10^. also established a type V (gastrosplenic trunk), was the most common variant of the coeliac trunk followed by a type II (hepatosplenic trunk) in the study population in Ankara hospital, Turkey.

Overall, the left renal artery showed a higher percentage of variant anatomy compared to the right renal artery in the current study. The most common variation of both the left and the right renal arteries was a type 1c (2 hilar arteries). Furthermore, this variant was seen more commonly in the left renal artery (9.1%) compared to the right renal artery (3.4%). This is in contrast to the study by Urgurel et al.^10^, which showed that variant anatomy of the right renal artery was more prevalent than the left renal artery in the study population in Ankara hospital, Turkey. Variations of both renal arteries were present in 2.4% of the study population.

The above illustrates that the most common variations of the coeliac trunk in this study was comparable to the findings of other studies conducted. The presence of variant anatomy in the renal arteries was more prevalent on the left compared to the right.

Concurrent variations of the coeliac trunk and the renal arteries were observed in 5% of the study population. These results show that multiple vascular variations may co-exist, confirming previous findings that when a vascular variation is found in one organ system, vascular variations in other organ systems should be sought. Although the presence of multiple variations in this study population was only 5%, it is important as the presence of multiple vascular variations may influence the surgical approach or surgical outcome in patients.

## Conclusion

Knowledge of anatomical variations of the coeliac trunk and renal arteries is of significant importance in the surgical approach and influences the subsequent surgical outcome in patients. It is therefore vital for radiologists to recognise, classify and report these anatomical variations. The most common coeliac trunk variations encountered in this study were comparable to other non-African populations. This illustrates that the most common variations of the coeliac trunk appear to be independent of factors such as race and ethnicity. Renal artery variations were more prevalent on the left than the right.

Furthermore, concurrent variations of the coeliac trunk and the renal arteries were observed in 5% of the study population. The presence of concurrent variations should be sought as the presence of multiple vascular variations may significantly influence the surgical approach chosen and aid in a reduction of surgical complications in patients.
